# Treatment persistence with aripiprazole once monthly: a 4-year follow-up

**DOI:** 10.1186/s12991-022-00416-z

**Published:** 2022-09-29

**Authors:** Andrea Fagiolini, Eugenio Aguglia, Andrea Ballerini, Gaetano Callista, Bernardo Carpiniello, Massimo Clerici, Giulio Corrivetti, Alessandro Cuomo, Pasquale De Fazio, Sergio De Filippis, Serafino De Giorgi, Arianna Goracci, Daniele La Barbera, Claudio Mencacci, Gino Montagnani, Giorgio Pigato, Jarno Vannucchi, Antonio Vita

**Affiliations:** 1 Division of Psychiatry, University of Siena, Viale Bracci 12, 53100 Siena, Italy; 2 University of Catania, Catania, Italy; 3 U Sod Di Psichiatria, AOU Careggi Firenze, Firenze, Italy; 4 UOSD S.P.D.C. P.O. Giulianova Asl Teramo, Teramo, Italy; 5 University of Cagliari, Cagliari, Italy; 6 University of Milano Bicocca, Milano, Italy; 7 ASL Salerno, EBRIS Foundation, Salerno, Italy; 8 University Magna Graecia, Catanzaro, Italy; 9 Neuropsychiatric Clinic Villa Von Siebenthal- Roma, Roma, Italy; 10 Department of Mental Health, ASL Lecce, Lecce, Italy; 11 University of Palermo, Palermo, Italy; 12 DSMD - Neuroscienze Asst Fatebenefratelli- Sacco, Milano, Italy; 13 Lundbeck Italia S.P.A, Milano, Italy; 14 University of Padova Medical Center, Padova, Italy; 15 Otsuka Pharmaceuticals, Milano, Italy; 16 University of Brescia, Brescia, Italy

**Keywords:** Aripiprazole, Long acting, Persistence, Maintenance, Adherence, Retention

## Abstract

**Objectives:**

Treatment persistence refers to the act of continuing a treatment as prescribed and reflects the patient's or doctor's judgment about efficacy, tolerability, and acceptability. In patients with schizophrenia, antipsychotic persistence is often poor, because of issues such as lack or loss of efficacy, side effects, and poor adherence, which is often related to the degree to which patients find the medication and overall intervention to be helpful, tolerable, fair, reasonable, appropriate, and consistent with expectations of treatment. Despite the poor antipsychotic persistence that has been reported to date in patients with schizophrenia, we previously observed a relatively high (86%) 6 months persistence with aripiprazole once-monthly (AOM) in a group of patients with schizophrenia, treated in the real world Italian clinical practice. The present study explores the longer term persistence with AOM, over a mean follow-up period of 48 months.

**Methods:**

This was a multicenter, retrospective, non-interventional follow-up study, aimed at evaluating the longer term persistence with AOM in a group of patients with schizophrenia who had already shown persistence over a period of at least 6 months. The study included 161 individuals who had participated in our previous study, where 86% of participating individuals had shown persistence with AOM for at least 6 months. Non-persistence was defined as discontinuing the medication for any reason. Baseline demographic and clinical characteristics of patients who continued AOM were then compared to those of patients who discontinued the medication.

**Results:**

Study subjects were predominantly male (64.4%) and their mean age was 39.7 (SD: 12.24). Treatment persistence with AOM was 69.6% and 112 out of 161 patients were still receiving AOM treatment at the last follow-up visit.

The mean duration of AOM treatment until the last recorded observation was 55.87 months (median 56.17, SD6.23) for the 112 persistent patients and 32.23 (median 28.68.SD 15.09) months for the 49 non-persistent individuals. The mean observation period for all patients (persistent and non-persistent) was 48.78 months (median 52.54, SD 14.64). For non-persistent subjects, the observation period ended with the discontinuation of AOM.

Subjects treated with AOM at 400 mg presented a 69.6% lower risk of all-cause treatment discontinuation when compared with patients treated with 300 mg (HR: 0.314; 95% confidence interval [CI] 0.162–0.608; *P* = 0.001). The main reasons for discontinuation were lack of efficacy (30.6%), patient/caregiver choice (18.4%), physician’s choice (16.3%), non-adherence (12.2%) and inconvenience (6.1%). Only 3 patients (6.1%) discontinued AOM for tolerability issues.

**Conclusions:**

In subjects with schizophrenia, who had already shown a 6 months persistence with AOM, a high number of patients (69.6%) continued to be persistent over a 4-year follow-up period. This may reflect a favourable profile of efficacy, tolerability, and acceptability. Larger and prospective studies are warranted to confirm our observations.

## Introduction

Antipsychotic persistence refers to the act of continuing an antipsychotic as prescribed and reflects the patient's or healthcare provider’s judgment about the efficacy, tolerability, acceptability, and perceived need for that medication [1]. Hence, causes of non-persistence to antipsychotics, include poor efficacy, poor tolerability, and poor adherence.

The Clinical Antipsychotic Trials of Intervention Effectiveness (CATIE) trial, demonstrated that only 371 (26%) of the 1432 patients who received at least one antipsychotic dose were persistent with their oral antipsychotic treatment over 18 months of treatment [10]. Long-acting injectable antipsychotics (LAI) improve adherence in patients with schizophrenia and several studies have shown their ability to reduce the rates of discontinuation, relapse, and hospitalization [1, 4, 6, 8, 14, 16].

We previously studied the 6 months persistence with aripiprazole once-monthly (AOM 400) in a group of 261 patients with schizophrenia recruited in the setting of the Italian National Public Health System [3] and found that 86% of study subjects were persistent with AOM for at least 6 months. A similar study evaluated the 6-month AOM persistence, in 91 patients with schizophrenia treated in Spain. 6 months after AOM initiation, 65 (71.4%) patients were persistent, whereas 26 (28.6%) were not [13]. Using a claims database, a recent study evaluated whether AOM can contribute to longer treatment persistence compared with oral aripiprazole (OA) in real-world clinical settings in Japan [9]. The Authors reported that patients treated with AOM group were significantly less likely to discontinue treatment than the OA group (adjusted HR 0.54, 95% confidence interval [CI] 0.43–0.68) and concluded that AOM was associated with longer treatment persistence than OA.

The present study evaluates the treatment persistence over a longer period (55.87 months for the 112 persistent patients and 32.23 months for the 49 non-persistent individuals, whose follow up was interrupted at the time of AOM discontinuation), in the same group of patients that participated in our previous 6-month persistence study [3]. We also evaluated the differences in clinical and demographic characteristics between patients who were or were not persistent with AOM.

## Methods

DOMINO study was a non-interventional, retrospective, observational, multicenter study involving patients diagnosed with schizophrenia and aimed at evaluating the rate of persistence with AOM over a 6-month period, along with the influence of baseline demographic and clinical variables on the likelihood to be persistent [3]. The present study (DOMINO II) reports on a longer term follow-up, over a mean period of 48.8 months from AOM initiation, involving the patients who were persistent for 6 months or longer in DOMINO study. Our primary objective was to evaluate the long-term persistence with AOM treatment, as defined as being still on AOM at the time of the last follow-up observation. The secondary objectives included the evaluation of efficacy and tolerability. The study was approved by the Ethical Committee/Institutional Review Board at each recruitment site. Written informed consent was obtained from all participants or their legal guardians. DOMINO study [3] was conducted between June 1st 2015 and July, 11th 2017. All patients presenting for a visit, who were at least 18 years of age, were diagnosed with schizophrenia, and had started ALAI (at least 1 injection) at least 6 months before the inclusion visit, were continuously recruited. Retrospective information from the start of A-LAI treatment (index date, baseline timepoint) until the follow-up visit (inclusion visit) was collected from A-LAI start to the follow-up visit (inclusion visit). The present DOMINO II study, extended the observation period to November 23, 2020.

The study was conducted between June 1, 2015 and November 23, 2020, in 16 clinical sites in Italy. All patients were at least 18 years and carried a diagnosis of schizophrenia, as established via a clinical interview conducted to verify the presence of the Diagnostic and Statistical Manual for Mental Disorders, 5th Edition (DSM 5) criteria.

Persistence was defined as receiving AOM at the last follow up visit and measured by time to all-cause AOM discontinuation and was assumed to reflect AOM’s efficacy, safety, and tolerability from both patient and treating health care provider (HCP)’s perspectives. Non-persistence was declared if the patient missed, for any reason (discontinuation, loss to follow up or skipped AOM doses), at least 2 consecutive or 3 non-consecutive injections of AOM. A missed dose of AOM was defined as a lapse of more than 45 days from the previous AOM injection. Electronic and paper clinical records were reviewed and treating HCP were interviewed as per the date of AOM interruption or discontinuation and reasons, when applicable.

### Statistical methods

The analyses were performed using Statistical Package for Social Science (SPSS-IBM, version 21.0 or later). Level of significance was set at a *p* value < 0.05 for two-tailed hypothesis. The “Full Analysis Set” (FAS) population was used to evaluate the primary objective. The FAS population included all patients persistent to AOM at the end of the DOMINO study observational period (6 months) for whom the information about the primary variables (long term persistence with AOM treatment) was available. The continuous variables were reported by sample statistics: *n* (number of observations), number of missing data, mean, standard deviation (SD), minimum, first quartile (Q1), median, third quartile (Q3), and maximum. The summary statistics mean, median, standard deviation, minimum, maximum, Q1 and Q3 were presented to one more decimal places as that used to collect the data.

Treatment persistence was defined as the proportion of patients who maintained the same medication (AOM) in a specific period. Treatment persistence might be considered as a proxy for treatment efficacy and safety, given that lack of efficacy or poor tolerability are among the main reasons for discontinuation. As in DOMINO study, non-persistence was declared if a patient missed 2 consecutive or 3 non-consecutive injections of AOM during the retrospective follow-up period. Treatment persistence was evaluated using Kaplan Meier survival method. Person-years at risk were computed from the beginning of AOM treatment to the point of treatment discontinuation, end of the study (last retrospective assessment, scheduled to be completed by the end of November 2020), or loss to follow-up, whichever occurred first. The event for “discontinuation” and the determination of RR was the interruption of AOM treatment. Patients lost to follow-up were considered as censored. The starting time for the Kaplan Meier analysis was the index date (date of AOM initiation).

Using a Cox Regression model demographic and clinical characteristics were evaluated for their effect on the long-term persistence to AOM. The variables included in the model were sourced from medical records or directly verified with the patients: age, gender, marital status, education, occupation, living situation and family support, number of previous acute episodes, schizophrenia, comorbidities, history of non-adherence in the 3 months prior to the index date, alcohol and/or drug abuse, reason to initiate AOM treatment, last antipsychotic treatment prior to AOM, CGI-S at index date and starting AOM dose.

Prior to the main analysis of the primary objective, univariate analyses (Chi Square tests, Student T tests or Mann–Whitney tests, as applicable) were performed to test the association between demographic and clinical characteristics of patients and long-term persistence with AOM (each baseline variable was compared between patients still in treatment at the end of the long-term follow-up period and patients that interrupted/discontinued the treatment before long term follow-up visit).

Univariate and multivariate Hazard Ratios (HR) with 95% Confidence Intervals (CIs) were calculated by regression model in which demographic and clinical characteristics were the independent variables and persistence was the dependent variable. The Cox Regression model produces a survival function that predict the probability of the discontinuation (event of interest) occurrence at a given time t for given predictor variable values. The shape of the survival function and the regression coefficients for the predictors were estimated from observed subjects. Cox’s semi-parametric model was used in the data analysis to explain the effect of explanatory variables on hazard rates.

#### Secondary objectives

The FAS population was used to evaluate all the secondary objectives. The analysis of the secondary objectives was essentially descriptive. Data were presented using summary statistics. All categorical variables were summarized in frequency and percentage. The continuous variables were reported by sample statistics: *n *(number of observations), number of missing data, mean, SD, minimum, Q1, median, Q3, and maximum. The summary statistics mean, median, standard deviation, minimum, maximum, Q1 and Q3 were presented to one more decimal places as that used to collect the data.

CGI-Severity score (index date, DOMINO inclusion visit date, long term follow-up visit date) was compared using the non-parametric Wilcoxon Signed-Rank test. The test is a non-parametric analogous of the one-sample *t* test. This test can be used to make inferences about a population mean or median, without requiring the assumption of normally distributed data. In addition, all CGI–severity score data collected for each patient after the index date, were analyzed descriptively.

## Results

Two-hundred-twenty-five (225) patients persistent to AOM at the end of the DOMINO study observational period were eligible to participate in this study. The same chronological inclusion order was followed, based on DOMINO patient log archived at each site. One-hundred-sixty-one subjects were included in this study. 64 patients did not participate, because four DOMINO sites declined to participate in DOMINO-II study or because of insufficient data quality. Study subjects (*n* = 161) were predominantly male (64.4%) and their mean age was 39.7 (SD: 12.24).

**Fig. 1:**
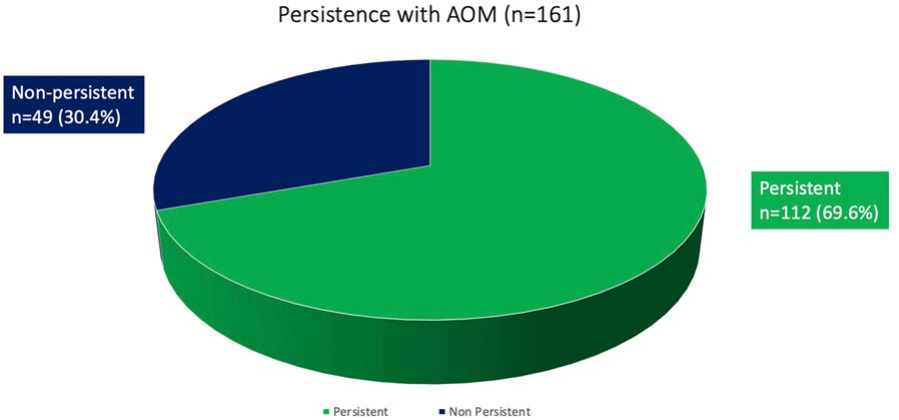
Persistence with AOM over a mean period of 48.78 months (*n* = 161)

Treatment persistence with AOM was 69.6% and 112 out of 161 patients were still receiving AOM treatment at the last follow-up visit (Fig. 1).

**Fig. 2:**
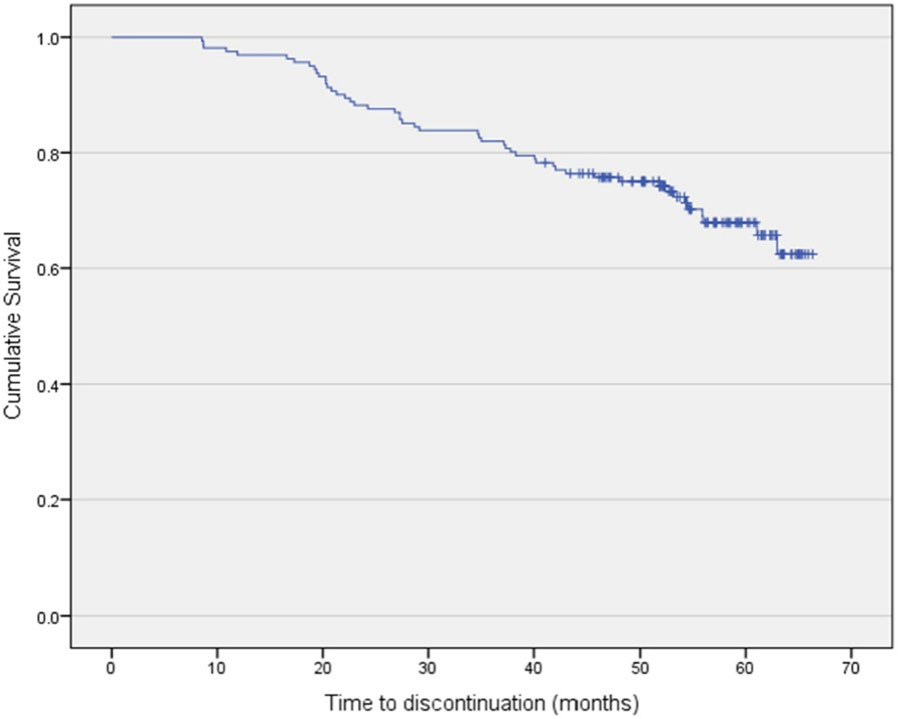
Time (months) to all-cause treatment discontinuation (Kaplan Meier)

The mean duration of treatment was 55.87 months (median 56.17, SD6.23) for persistent patients, and 32.23 (median 28.68.SD 15.09) months for non-persistent patients (*P*< 0.001). For non-persistent patients, the follow-up ended at the time of AOM discontinuation. The mean observation period following AOM initiation in the entire group of patients (persistent and non-persistent) was 48.78 months (median 52.54, SD 14.64). Figure 2 reports the time (months) to all-cause treatment discontinuation (Kaplan Meier).

No significant difference was found between persistent and non-persistent patients for: age at AOM initiation, age at the last follow up visit, gender, education, marital status, occupation, age at first schizophrenia diagnosis, time since schizophrenia onset, number of relapses in the previous 5 years, alcohol or drug abuse.

**Fig. 3:**
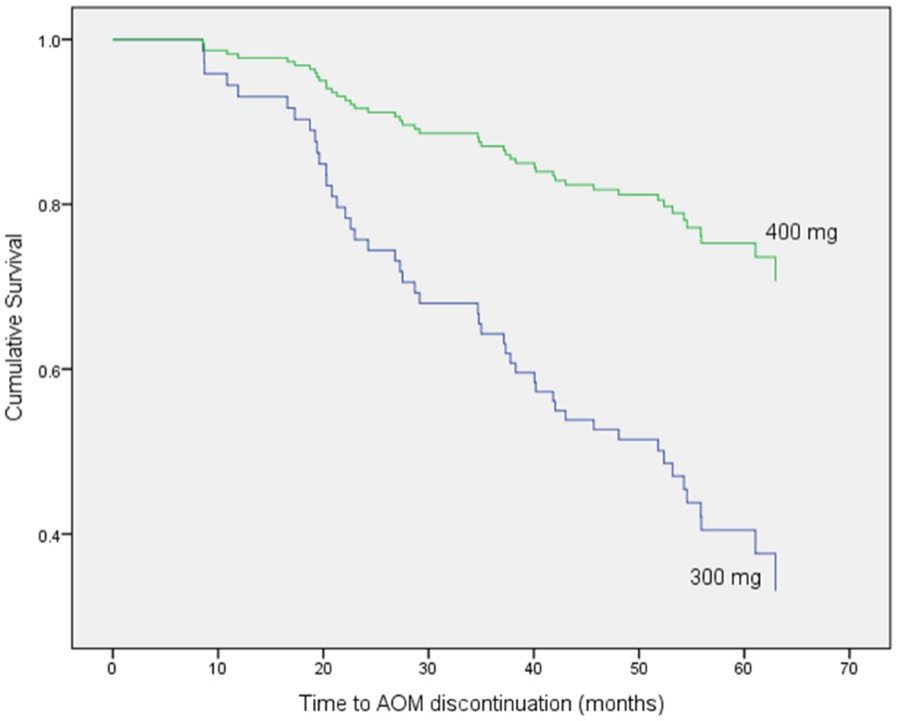
Time (months) to all-cause treatment discontinuation (Kaplan Meier) by. Starting AOM dose

**Fig. 4:**
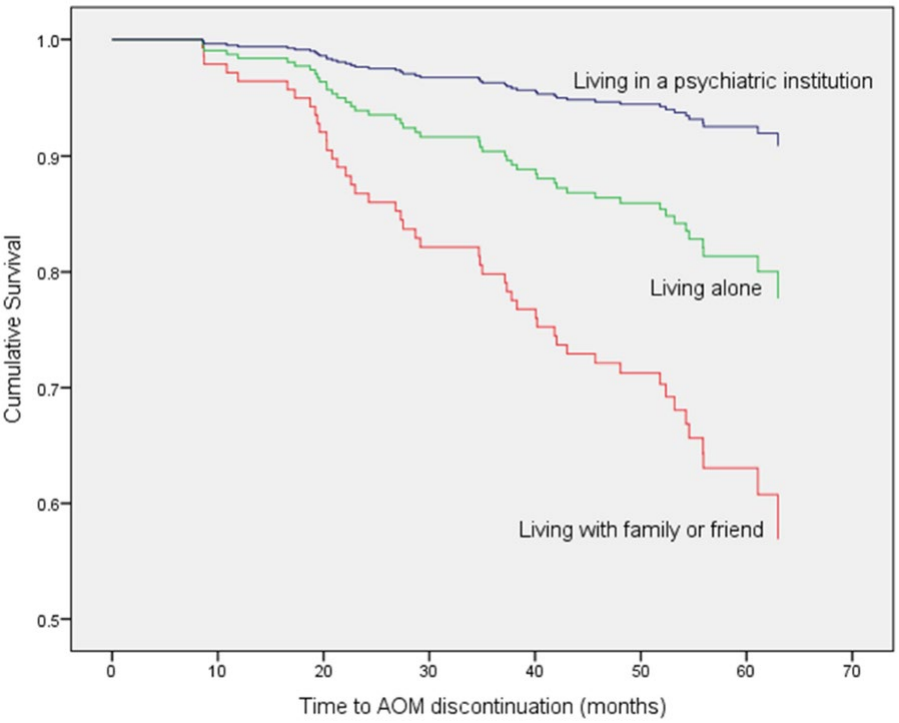
Time (months) to all-cause treatment discontinuation (Kaplan Meier) by Living situation and family support

The multivariate model showed that:

1. Patients treated with an AOM dose of 400 mg presented a 69.6% lower risk of all-cause treatment discontinuation when compared with patients treated with a dose of 300 mg (HR: 0.314; 95% confidence interval [CI] 0.162–0.608; *P* = 0.001) (Fig. 3).
2. Patients who lived alone presented a 55.3% lower risk of discontinuation when compared with patients who lived with family or friends but the difference was not statistically significant (HR: 0.447; 95% confidence interval [CI] 0.176–1.135; *P* = 0.090)
3. Patients who lived in a psychiatric assisted living facility presented an 83.0% lower risk of discontinuation when compared with patients who lived alone, or with their family (HR: 0.170; 95% confidence interval [CI] 0.023–1.236; *P* = 0.080), but the difference was not statistically significant (Fig. 4).

Tables 1, 2 report the Clinical Global Impression scores at intake and at the last follow-up observation, and the difference between persistent and non-persistent individuals.

**Table 1:** CGI scores in persistent and non-persistent patients, at index visit

Severity scale (CGI-S) at index visit				
Wilcox on rank-sum test *p* value = 0.631 (subjects ‘‘not accessed’’ were excluded)		Persistence with AOM treatment		Total (*N* = 161)
		Yes (*N* = 112)	No (*N* = 49)	
CGI-S				
Not assessed	Count	34	5	39
1 = Normal	Count	1	1	2
	%	1.3	2.3	1.6
2 = Borderline mentally ill	Count	1	1	2
	%	1.3	2.3	1.6
3 = Mildly ill	Count	7	6	13
	%	9.0	13.6	10.7
4 = Moderately ill	Count	21	9	30
	%	26.9	20.5	24.6
5 = Marked ill	Count	28	15	43
	%	35.9	34.1	35.2
6 = Severely ill	Count	13	11	24
	%	16.7	25.0	19.7
7 = Among the most extremely ill patients	Count	7	1	8
	%	9.0	2.3	6.6
Total	Count	78	44	122
	%	100.0	100.0	100.0

**Table 2:** CGI scores in persistent and non-persistent patients, at the last follow-up Visit

Severity scale (CGI-S) at last available follow-up visit				
Wilcox on rank-sum test *p* value = 0.631 (subjects ‘‘not accessed’’ were excluded)		Persistence with AOM treatment		Total (*N* = 161)
		Yes (*N* = 112)	No (*N* = 49)	
CGI-S				
Not assessed	Count	4	2	6
1 = Normal	Count	0	0	0
	%	0.0	0.0	0.0
2 = Borderline mentally ill	Count	16	9	25
	%	14.8	19.1	16.1
3 = Mildly ill	Count	12	5	17
	%	11.1	10.6	11.0
4 = Moderately ill	Count	37	10	47
	%	34.3	21.3	30.3
5 = Marked ill	Count	37	8	45
	%	34.3	17.0	29.0
6 = Severely ill	Count	6	9	15
	%	5.6	19.1	9.7
7 = Among the most extremely ill patients	Count	0	6	6
	%	0.0	12.8	3.9
Total	Count	108	47	155
	%	100.0	100.0%	100.0

CGI score improved from baseline to the last follow up visit in 59 (49.2%) of the 120 patients whose CGI was recorded both at intake and at the follow up visit. CGI score worsened in 24 (20.0%) and did not change in the remaining 37 (30.8%) patients.

The percentage of patients whose CGI worsened was higher (34.9% vs. 11.7%) in the non-persistent patients.

Forty-nine patients discontinued the medication during the observed follow-up period. The main reasons for discontinuation were lack of efficacy (30.6%), patient/caregiver choice (18.4%), physician’s choice (16.3%), non-adherence (12.2%) and inconvenience (6.1%). Only 3 patients (6.1%) discontinued AOM for tolerability issues. Three patients died during the period covered by the study. In none of the three cases, the death was related to AOM.

## Discussion

Treatment persistence reflects the patient's or clinician’s judgment about the prescribed medication. In patients with schizophrenia, treatment persistence is often undermined by lack or loss of efficacy, poor tolerability, non- adherence or poor adherence to treatment [2, 5, 7, 11, 14, 15]. The Clinical Antipsychotic Trials of Intervention Effectiveness (CATIE) trial evaluated 1432 patients with schizophrenia, who were randomly assigned to receive olanzapine, perphenazine, quetiapine, risperidone, or ziprasidone for up to 18 months. The study demonstrated that 74% of patients (1061 out of 1432) discontinued the study medication before 18 months: 64% of those assigned to olanzapine, 75% of those assigned to perphenazine, 82% of those assigned to quetiapine, 74% of those assigned to risperidone, and 79% of those assigned to ziprasidone. Hence, only 26% of patients were persistent for 18 months, with their prescribed antipsychotic.

While the comparative efficacy of antipsychotic medications is sensitive to research design, long-acting antipsychotics have frequently displayed significant advantages over the oral formulations in real world, observational, studies [8]. Results from randomized placebo-controlled trials are less consistent but this may be at least partially due to the possibility that the research procedures and settings of randomized, placebo controlled trials, reduces the risk of non-adherence to oral medications [7].

Also, patients who agree to participate in a randomized and placebo-controlled clinical trial are usually more likely to be adherent to the prescribed medication, compared with patients that are treated in the real-world setting. In fact, the structured setting of a randomized and placebo-controlled trial, likely reduces the rates of non-adherence, given its relatively intense monitoring and assessments schedules and, when allowed, the presence of incentives and reimbursements. Moreover, in real world, observational, studies it is possible to add oral antipsychotics or psychosocial interventions to antipsychotics in case of patients' worsening.

AOM combines the advantages of depot antipsychotics, for instance in terms of improving adherence, with a proven efficacy and a relatively benign tolerability profile [12].

That we observed that 69.6% of patients were still persistent after 48 months of AOM treatment, with only 30.4% of AOM patients that did not continue the medication for at least 48 months, confirms our hypothesis that AOM is a medication that is associated with a high persistence rate, not only for the short period (as demonstrated in our previous 6-month persistence study) but also for the longer term. We believe that this may be correlated with the relatively benign tolerability profile of AOM, along with the high degree of adherence usually associated with long acting antipsychotics, and the efficacy as a long term preventative medication [3, 7, 13]. Interestingly, we noted that patients treated with an AOM dose of 400 mg presented a 69.6% lower risk of all-cause treatment discontinuation when compared with patients treated with a dose of 300 mg (HR: 0.314; 95% CI 0.162–0.608; *P* = 0.001), this suggesting the possibility that the higher dose (400 mg) might have greater efficacy than 300 mg. However, the reasons why patients were on 300 mg was not recorded, and therefore, we cannot exclude that patients were prescribed the 300 mg dose, because they were more sensitive to side effects, which could increase the likelihood of a following discontinuation.

This study has several limitations, including the following: (1) most patients were not on monotherapy and could receive oral antipsychotics or psychosocial interventions added to AOM in case of patients' worsening; (2) the concomitant medications could be started, discontinued or modified during our observation period; (3) patients who were taking AOM at the time of study entry may have been less likely to be missed by error from recruitment; (4) the study was multicenter but all patients were recruited in a single Country (Italy), and therefore, the results may not be generalizable; (5) no comparison group, of patients treated with other antipsychotics, was present; (6) one inclusion criteria for the study was that patients already had AOM treatment persistence for 6 months throughout DOMINO study; as tolerability issues often arise at the beginning of treatment, this might represent a selection bias favoring overestimation of AOM treatment persistence.

## Conclusions

In DOMINO study, 86% of patients with schizophrenia were persistent to AOM for at least 6 months. This study shows 69% of these patients, i.e., those that were persistent for at least 6 months in DOMINO study, continued to be persistent for a much longer period of time (mean follow-up period for persistent patients: 55, 87 weeks). The high persistence rate, likely indicates a good efficacy and tolerability profile of AOM in the long term. Larger and prospective studies are warranted to confirm our observations.

## Data Availability

All data are stored by Otsuka Pharmaceutical Italy S.r.l. and is available for regulatory agencies.

## Abbreviation

AOM: Aripiprazole once-monthly
